# Effect of epidermal growth factor receptor gene polymorphisms on prognosis in glioma patients

**DOI:** 10.18632/oncotarget.10666

**Published:** 2016-07-18

**Authors:** Bin Li, Wenhui Zhao, Jingjie Li, Mengdan Yan, Zhilan Xie, Yuanyuan Zhu, Chao Chen, Tianbo Jin

**Affiliations:** ^1^ School of Life Sciences, Northwest University, Xi'an, Shaanxi, 710069, China; ^2^ National Engineering Research Center for Miniaturized Detection Systems, Xi'an, Shaanxi, 710069, China; ^3^ Department of Anesthesiology, Shaanxi Provincial Tumor Hospital, Xi'an, Shaanxi, 710061, China; ^4^ Xi'an Tiangen Precision Medical Institute, Xi'an, Shaanxi 710075, China

**Keywords:** EGFR, glioma, polymorphism, prognosis

## Abstract

Previous studies suggested that single nucleotide polymorphisms (SNPs) in epidermal growth factor receptor (*EGFR*) are associated with risk of glioma. However, the associations between these SNPs and glioma patient prognosis have not yet been fully investigated. Therefore, the present study was aimed to evaluate the effects of *EGFR* polymorphisms on the glioma patient prognosis. We retrospectively evaluated 269 glioma patients and investigated associations between *EGFR* SNPs and patient prognosis using Cox proportional hazard models and Kaplan-Meier curves. Univariate analysis revealed that age, gross-total resection and chemotherapy were associated with the prognosis of glioma patients (*p* < 0.05). In addition, four *EGFR* SNPs (rs11506105, rs3752651, rs1468727 and rs845552) correlated with overall survival (OS) (Log-rank *p* = 0.011, 0.020, 0.008, and 0.009, respectively) and progression-free survival PFS (Log-rank *p* = 0.026, 0.024, 0.019 and 0.009, respectively). Multivariate analysis indicated that the rs11506105 G/G genotype, the rs3752651 and rs1468727 C/C genotype and the rs845552 A/A genotype correlated inversely with OS and PFS. In addition, OS among patients with the rs730437 C/C genotype (*p* = 0.030) was significantly lower OS than among patients with A/A genotype. These data suggest that five EGFR SNPs (rs11506105, rs3752651, rs1468727, rs845552 and rs730437) correlated with glioma patient prognosis, and should be furthered validated in studies of ethnically diverse patients.

## INTRODUCTION

Glioma that arises from glial or precursor cells is one of the most common and aggressive malignant primary intracranial brain tumors. The incidence rate 6.02 per 100,000, particularly among adults [1]. Glioma accounts for approximately 30% of all brain and central nervous system tumors and 80% of all malignant brain tumors [2]. It is one of the primary causes of cancer-related deaths worldwide and of refractory cancer in the field of neurosurgery. Gliomas are classified as astrocytomas, oligodendrogliomas, oligoastrocytomas and glioblastomas based on cellular lineage. The World Health Organization (WHO) classifies gliomas into four clinical grades: I, pilocytic astrocytomas; II, diffuse low grade gliomas; III, anaplastic gliomas; and IV, glioblastoma [3].

Gliomas are characterized by extensive vasculature, rapid growth, and a short disease course. Because gliomas typically invade the perivascular spaces within the brain tissue resulting in irregular borders, they are difficult to completely resect surgically [4]. Therefore, patients have relatively high postoperative recurrence rates and short survival times. Despite significant improvement in glioma diagnostics and therapeutics (e.g., surgery, radiotherapy and chemotherapy), the prognosis of glioma patients is still dismal compared to patients with other types of brain tumors. For example, the median survival time (MST) of patients with glioblastoma ranges from 12 to 15 months and the majority of patients die within 2 years of diagnosis. At present, the precise etiology and pathogenic mechanisms underlying glioma development and progression are unknown.

Several clinical factors affect the prognosis of glioma patients. These include age; gender; preoperative symptoms; preoperative disease duration; preoperative Karnofsky Performance Status (KPS) score; tumor grade, size, range and location; and histological grade. There are also several factors related to treatment that can affect patient prognosis including the surgical method, extent of surgical resection, duration of surgery, radiotherapy and chemotherapy. Most gliomas result from the combined action of environmental factors and inherited genetic variations. Many studies have suggested that genetic polymorphisms in the epidermal growth factor receptor (*EGFR*) are associated with risk of glioma [5–7], breast cancer [8], colorectal adenoma and colorectal cancer [9], and non-small cell lung cancer [10].

The *EGFR* gene is located on chromosome 7p12-13 and encodes for a 170 kD transmembrane receptor tyrosine kinase that is expressed on the surface of epithelial cells [11]. What is more, it is the first receptor discovered to possess tyrosine kinase activity and to be sequenced. Previous have demonstrated that activation of the EGFR signaling pathway contributes to many biological processes including cell survival, proliferation, apoptosis, differentiation, cell cycle progression, invasion, metastasis and angiogenesis [12, 13], all of which are associated with tumor progression. EGFR signaling is initiated by ligand binding to the extracellular ligand-binding domain, which initiates receptor dimerization and tyrosine auto-phosphorylation, resulting in receptor activation [14]. Amplification and/or overexpression of *EGFR* have been observed in approximately 50% of malignant gliomas [15] compared to approximately 10 to 26% of anaplastic astrocytomas [16]. *EGFR* amplification was associated with worse outcomes in glioblastoma patients [17]. However, the exact pathogenesis and biological mechanisms by which polymorphisms in *EGFR* contribute to glioma development are unclear.

Although previous association studies demonstrated that genetic polymorphisms in *EGFR* were associated with glioma risk, few studies focused on the effects of these alterations on glioma patient prognosis. We hypothesized that SNPs in *EGFR* could impact the prognosis of glioma patient. To test this hypothesis, we screened and genotyped eight SNPs in *EGFR* and evaluated the associations between these SNPs and the prognosis of glioma patients in a Chinese population.

## RESULTS

### Patient characteristics and clinical data

A total of 269 glioma patients were analyzed in this study: 160 astrocytoma patients, 19 ependymoma, 9 oligodendroglioma, 31 oligodendrocyte astrocytoma, 42 glioblastoma and 8 with other types of glioma. The characteristics and detailed clinical data for the patients with glioma as well as 160 astrocytoma patients (WHO grade I–IIΙ) are summarized in Table 1. Among 269 glioma patients, there were 145 (53.9%) men and 124 (46.1%) women. There were 116 patients < 40 years of age and 153 ≥ 40 years of age. A total of 18 (6.7%) patients were classified as WHO grade I, 129 (48.0%) as WHO grade II, 72 (26.8%) as WHO grade III, and 50 (18.6%) as WHO grade IV. Gamma knife radiotherapy was administered to 176 (65.4%) patients and conformal radiation therapy was administered to 69 (25.7%) patients. Finally, 104/269 (38.7%) patients received chemotherapy. At the time of the last follow-up, 249 (92.6%) glioma patients had died. The results of the genotyping for the eight SNPs are shown in Supplementary Figure 1.

**Table 1:** Characteristics of glioma and astrocytoma subjects

Variable	Classification	Glioma		Astrocytoma	
		No. of Patients	Percent	No. of Patients	Percent
Gender	Male	145	53.9%	88	55.0%
	Female	124	46.1%	72	45.0%
Age (years)	< 40	116	43.1%	66	41.3%
	≥ 40	153	56.9%	94	58.8%
WHO grade	WHO I	18	6.7%	18	11.3%
	WHO II	129	48.0%	78	48.8%
	WHO III	72	26.8%	64	40.0%
	WHO IV	50	18.6%	-	-
Extent of resection	GTR	184	68.4%	111	69.4%
	STR or NTR	85	31.6%	49	30.6%
Radiotherapy	GK	176	65.4%	106	66.3%
	CRT	69	25.7%	41	25.6%
	No	24	8.9%	13	8.1%
Chemotherapy	Platinum	56	20.8%	37	23.1%
	Nimustine	32	11.9%	14	8.8%
	Temozolomide	16	5.9%	7	4.4%
	No	165	61.3%	102	63.8%
Survival condition	Survival	11	4.1%	6	3.8%
	Lost	9	3.3%	7	4.4%
	Death	249	92.6%	147	91.9%
Progress	Yes	10	3.7%	6	3.8%
	No	255	94.8%	152	95.0%
	Missing system	4	1.5%	2	1.3%

WHO: World Health Organization; GTR: Gross-total resection; NTR: Near-total resection;

STR: Sub-total resection; GK: Gamma knife; CRT: Conformal radiation therapy.

### Univariate analysis

Clinical factors including gender, age, WHO grade, extent of resection, radiotherapy and chemotherapy were assessed in a univariate analysis (Tables 2 and 3). We determined that age ≥ 40 was a hazardous factor with a 1.30-fold (Log-rank *p* = 0.025, hazard ratio [HR] = 1.302, 95% confidence interval [CI] = 1.010–1.678, *p* = 0.041) and 1.29-fold (Log-rank *p* = 0.025, HR = 1.290, 95% CI = 1.004–1.659, *p* = 0.047) increased risk of death on overall survival (OS) and progression-free survival (PFS) in glioma patients, respectively. The MSTs was 10 and 8 months, and the 3-year survival rates were 4.2% and 2.6% of the OS and PFS, respectively (Table 2). In contrast, the extent of resection (gross-total resection) and chemotherapy were protective against mortality factors. Gross-total resection was associated with a 35.5% (Log-rank *p* = 0.000, HR = 0.645, 95% CI = 0.492–0.845, *p* = 0.001) and 38.6% (Log-rank *p* = 0.000, HR = 0.614, 95% CI = 0.468–0.806, *p* = 0.000) decrease in mortality hazard, the MSTs were 11and 8 months, 3-years survival rates were 8.9% and 4.9% of the OS and PFS in glioma patients, respectively. Treatment with chemotherapy was also associated with a reduced risk of death measured by OS and PFS in glioma patients (Log-rank *p* =0.001, HR = 0.660, 95% CI = 0.506–0.860, *p* = 0.002; Log-rank *p* = 0.019, HR = 0.755, 95% CI = 0.580–0.984, *p* = 0.038, respectively) (Table 2). The gross-total resection and chemotherapy were also positive factors in astrocytoma patients with OS (Log-rank *p* = 0.003, HR = 0.614, 95% CI = 0.431–0.875, *p* = 0.007; Log-rank *p* = 0.020, HR = 0.705, 95% CI = 0.498–0.997, *p* = 0.048, respectively), and the MSTs were11and12 months respectively. Gross-total resection was associated with the PFS in astrocytoma patients (Log-rank *p* = 0.000, HR = 0.556, 95% CI = 0.389–0.794, *p* = 0.001), with an 8 month MST (Table 3). nNo significant correlations were identified between gender, WHO grade, or radiotherapy and prognosis the of glioma and astrocytoma patients as measured by OS and PFS.

**Table 2:** Univariate analysis of the impact of clinical factors on glioma patient OS and PFS

Variable	Classification	OS						PFS					
		No. of patients/events	1/3-(year) SR (%)	MST	Log-rank *p*	HR(95%CI)	*p*	No. of patients/events	1/3-(year) SR (%)	MST	Log-rank *p*	HR(95%CI)	*p*
Gender	Male	145/135	25.5/5.3	11		1		143/138	16.1/3.5	8		1	
	Female	124/114	35.5/7.9	11	0.522	0.928(0.723-1.192)	0.559	122/117	15.6/4.1	8	0.579	0.940(0.734-1.203)	0.622
Age(years)	<40	116/103	36.2/9.5	12		1		113/107	20.4/5.3	8		1	
	≥40	153/146	25.5/4.2	10	0.025	1.302(1.010-1.678)	0.041	152/148	12.5/2.6	8	0.025	1.290(1.004-1.659)	0.047
WHO grade	I-II	147/133	32.0/8.6	12		1		145/139	16.6/4.1	8		1	
	III-IV	122/116	27.9/4.6	10	0.127	1.194(0.930-1.532)	0.164	120/116	15.0/3.3	8	0.245	1.139(0.890-1.458)	0.302
Extent of resection	STR or NTR	85/84	17.6/1.2	10		1		82/81	12.0/−	8		1	
	GTR	184/165	35.9/8.9	11	0.000	0.645(0.492-0.845)	0.001	183/174	22.4/4.9	8	0.000	0.614(0.468-0.806)	0.000
Radiotherapy	No	24/22	33.3/8.3	8		1		21/21	9.5/−	6		1	
	CRT	69/60	20.3/11.8	9		0.856(0.523-1.402)	0.536	68/61	16.2/10.3	7		1.060(0.641-1.752)	0.821
	GK	176/167	33.5/5.2	11	0.681	0.834(0.534-1.303)	0.426	176/173	16.5/1.7	8	0.834	0.976(0.619-1.539)	0.918
Chemotherapy	No	165/158	24.1/2.7	9		1		164/164	15.9/−	7		1	
	Yes	104/91	39.4/12.7	12	0.001	0.660(0.506-0.860)	0.002	101/91	15.8/9.9	8	0.019	0.755(0.580-0.984)	0.038

OS: Overall survival; PFS: Progression free survival; WHO: World Health Organization; GTR: Gross-total resection; NTR: Near-total resection;

STR: Sub-total resection; CRT: Conformal radiation therapy; GK: Gamma knife; SR: Survival rate; MST: Median survival time (months)

HR: Hazard ratio; 95% CI: 95% Confidence interval

Log-rank *p* values were calculated from Chi-Square test

*p* values were calculated from Wald test

*p* < 0.05 indicates statistical significance

**Table 3:** Univariate analysis of the impact of clinical factors on astrocytoma patient OS and PFS

Variable	Classification	OS						PFS					
		No. of patients/events	1/3-(year) SR (%)	MST	Log-rank *p*	HR(95%CI)	*p*	No. of patients/events	1/3-(year) SR (%)	MST	Log-rank *p*	HR(95%CI)	*p*
Gender	Male	88/80	26.1/6.8	11		1		87/84	18.4/3.4	8		1	
	Female	72/67	31.9/6.3	11	0.769	1.045(0.755-1.447)	0.789	71/68	11.3/4.2	8	0.629	1.072(0.778-1.478)	0.669
Age(years)	<40	66/58	31.8/8.6	11		1		65/62	16.9/4.6	8		1	
	≥ 40	94/89	26.6/3.2	10	0.184	1.227(0.880-1.711)	0.227	93/90	14.0/3.2	8	0.140	1.243(0.896-1.723)	0.192
WHO grade	I-II	96/86	28.1/4.2	11		1		95/91	14.7/4.2	8		1	
	III	64/61	29.7/3.9	11	0.612	1.081(0.778-1.501)	0.643	63/61	15.9/3.2	8	0.959	1.008(0.728-1.394)	0.964
Extent of resection	STR or NTR	49/49	16.3/−	9		1		48/48	0/−	7		1	
	GTR	111/98	34.2/9.6	11	0.003	0.614(0.431-0.875)	0.007	110/104	21.8/5.5	8	0.000	0.556(0.389-0.794)	0.001
Radiotherapy	No	13/11	46.2/−	12		1		14/11	18.2/−	11		1	
	CRT	41/37	14.6/8.8	9		1.475(0.749-2.905)	0.261	41/38	12.2/7.3	7		1.700(0.861-3.355)	0.126
	GK	106/99	32.1/2.8	11	0.334	1.183(0.634-2.209)	0.598	106/103	16.0/2.8	8	0.163	1.326(0.709-2.480)	0.376
Chemotherapy	No	102/96	23.5/−	9		1		101/101	15.8/−	7		1	
	Yes	58/51	37.9/11.4	12	0.029	0.705(0.498-0.997)	0.048	57/51	14.0/10.5	8	0.137	0.794(0.562-1.119)	0.187

OS: Overall survival; PFS: Progression free survival; WHO: World Health Organization; GTR: Gross-total resection; NTR: Near-total resection;

STR: Sub-total resection; CRT: Conformal radiation therapy; GK: Gamma knife; SR: Survival rate; MST: Median survival time(months)

HR: Hazard ratio; 95% CI: 95% Confidence interval

Log-rank *p* values were calculated using the Chi-Square test

*p* values were calculated using the Wald test

*p* < 0.05 indicates statistical significance

**Figure 1:**
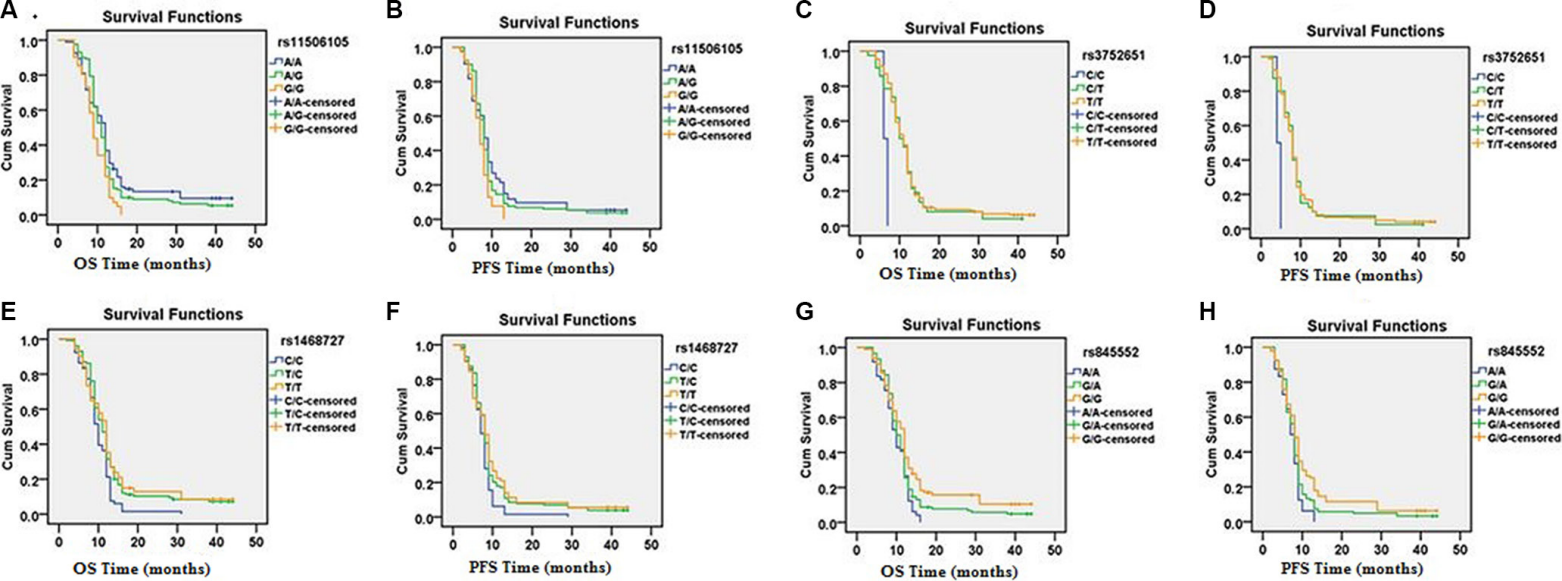
Kaplan-Meier curves for OS and PFS for patients with different genotypes corresponding to four *EGFR* SNPs (rs11506105, rs3752651, rs1468727 and rs845552)

According to Log-rank tests and Cox regression analysis, four of the eight SNPs evaluated in *EGFR* showed statistically significantly correlations with OS and PFS (Table 4). Kaplan-Meier curves of OS and PFS for the different genotypes of the four SNPs are shown in Figure 1A–1H. Interestingly, the G/G genotype of rs11506105 was correlated with poor prognosis in glioma patients as measured by OS and PFS (HR = 1.687, 95% CI = 1.156–2.462, *p* = 0.007; HR = 1.594, 95% CI = 1.087–2.337, *p* = 0.017, respectively). Similar results were observed for the C/C genotype of rs3752651, which was significantly associated with increased OS and PFS (HR = 5.725, 95% CI = 1.390–23.584, *p* = 0.016; HR = 5.445, 95% CI = 1.325–22.372, *p* = 0.019, respectively), relative to the T/T genotypes. The C/C genotype ofrs1468727 had a negative effect on OS and PFS compared to the common T/T genotype, (HR = 1.564, 95% CI = 1.103–2.218, *p* = 0.012; HR = 1.497, 95% CI = 1.057–2.120, *p* = 0.023, respectively). Furthermore, the A/A genotype of rs845552 was inversely correlated with OS (HR = 1.636, 95% CI = 1.144–2.339, *p* = 0.007) and PFS (HR = 1.628, 95% CI = 1.137–2.330, *p* = 0.008) in glioma patients. No significant associations were identified between the eight EGFR SNPs analyzed and OS or PFS in astrocytoma patients.

**Table 4:** Univariate analysis of the association between eight SNPs in *EGFR* and glioma patient OS and PFS

SNP-ID	Region	Genotype	OS						PFS					
			No. of patients/events	1 / 3(year) SR (%)	MST	Log-rank *p*	HR(95%CI)	*p*	No. of patients/events	1 / 3(year) SR (%)	MST	Log-rank *p*	HR(95%CI)	*p*
rs17172432	Intron 1	T/T	222/206	30.6/6.1	11	0.661	1		219/211	16.4/3.7	8	0.738	1	
		C/T	45/41	28.9/5.7	10		1.030(0.736-1.440)	0.864	44/42	13.6/4.5	8		0.992(0.712-1.382)	0.963
		C/C	2/2	-	9		1.817(0.449-7.354)	0.403	2/2	-	6		1.655(0.410-6.685)	0.480
rs4947492	Intron 1	A/A	108/98	32.4/6.5	11	0.402	1		108/104	16.7/3.7	8	0.481	1	
		G/A	132/122	43.9/6.8	11		1.049(0.804-1.369)	0.725	129/123	16.3/4.7	8		0.980(0.755-1.273)	0.882
rs12718945		G/G	29/29	24.1/−	10		1.298(0.856-1.967)	0.220	28/28	10.7/−	7		1.227(0.807-1.868)	0.339
	Intron 1	G/G	109/99	33.0/6.4	12	0.395	1		109/105	16.5/3.7	8	0.474	1	
		G/T	128/118	28.9/7.0	11		1.050(0.804-1.372)	0.720	125/119	16.8/4.8	8		0.983(0.756-1.278)	0.896
		T/T	29/29	24.1/−	10		1.300(0.858-1.970)	0.215	28/28	10.7/−	7		1.233(0.811-1.876)	0.327
rs730437	Intron 4	A/A	87/77	36.8/8.2	12	0.051	1		85/81	20.0/4.7	8	0.145	1	
		C/A	136/127	27.9/5.6	11		1.113(0.838-1.479)	0.459	136/131	14.7/3.7	8		1.090(0.825-1.440)	0.543
		C/C	46/45	23.9/2.2	9		1.513(1.045-2.190)	0.028	44/43	11.4/2.3	7		1.390(0.958-2.017)	0.083
rs11506105	Boundary	A/A	95/83	36.8/9.6	12	0.011	1		93/88	21.5/5.4	8	0.026	1	
		A/G	131/123	27.5/5.4	11		1.143(0.864-1.512)	0.348	131/126	14.5/3.8	8		1.112(0.846-1.462)	0.448
		G/G	41/41	22.0/−	9		1.687(1.156-2.462)	0.007	39/39	7.7/−	7		1.594(1.087-2.337)	0.017
rs3752651	Intron 13	T/T	224/207	29.9/6.3	11	0.020	1		222/213	16.7/4.1	8	0.024	1	
		C/T	42/39	31.0/4.1	10		1.039(0.738-1.463)	0.826	40/39	12.5/2.5	8		1.033(0.734-1.454)	0.851
		C/C	2/2	0/−	6		5.725(1.390-23.584)	0.016	2/2	-	4		5.445(1.325-22.372)	0.019
rs1468727	Intron 13	T/T	71/63	35.2/8.6	12	0.008	1		71/67	21.1/5.6	8	0.019	1	
		T/C	130/119	31.5/8.4	11		1.062(0.783-1.443)	0.698	128/123	17.2/3.9	8		1.066(0.791-1.436)	0.676
		C/C	66/66	21.2/0	10		1.564(1.103-2.218)	0.012	64/64	6.3/−	7		1.497(1.057-2.120)	0.023
rs845552	Intron 19	G/G	97/84	37.1/10.5	12	0.009	1		95/89	25.3/6.3	8	0.009	1	
		G/A	122/115	25.4/5.8	10		1.307(0.985-1.734)	0.064	121/117	12.4/3.3	8		1.279(0.968-1.689)	0.083
		A/A	49/49	26.5/−	10		1.636(1.144-2.339)	0.007	48/48	6.3/−	7		1.628(1.137-2.330)	0.008

SR: Survival rate; MST: Median survival time(months)

HR: Hazard ratio; 95% CI: 95% Confidence interval

Log-rank *p* values were calculated using the Chi-Square test

*p* values were calculated using the Wald test

*p* < 0.05 indicates statistical significance

### Multivariate analysis

After adjusting for the various clinical factors, multivariate Cox regression analysis demonstrated that the SNP genotype was an independent prognostic factor for OS and PFS. We identified significant correlations between five SNPs in *EGFR* (rs11506105, rs3752651, rs1468727, rs845552 and rs730437) and the prognosis of glioma patients (Table 5). The G/G genotype of rs11506105 was correlated with worse OS and PFS in glioma patients (adjusted HR = 1.680, 95% CI = 1.145–2.466, *p* = 0.008; adjusted HR = 1.542, 95% CI = 1.047–2.272, *p* = 0.028, respectively). Moreover, the C/C genotype of rs3752651 was prominently associated with a 5.313-fold (adjusted HR = 5.313, 95% CI = 1.279–22.074, *p* = 0.022) and 5.847-fold (adjusted HR = 5.847, 95% CI = 1.414–24.179, *p* = 0.015) risk of death as measured by OS and PFS, respectively. A very similar trend was observed for the C/C genotype of rs1468727, which had a significant impact on OS and PFS (adjusted HR = 1.650, 95% CI = 1.158–2.351, *p* = 0.006; adjusted HR = 1.487, 95% CI = 1.049–2.108, *p* = 0.026, respectively). In comparison to those patients with the genotype G/G of rs845552, the genotype A/A was associated with an increase in mortality hazard of borderline statistical significance as measured by OS and PFS (adjusted HR = 1.614, 95% CI = 1.127–2.312, *p* = 0.009; adjusted HR = 1.580, 95% CI = 1.104–2.270, *p* = 0.013, respectively). In addition, we found that the C/C genotype of rs730437 was associated with reduced OS compared to the A/A genotype (adjusted HR = 1.513, 95% CI = 1.041–2.201, *p* = 0.030). However, we did not identify an association between theses SNPs in *EGFR* and either OS and PFS in astrocytoma patients.

**Table 5:** Multivariate analysis of the associations between eight SNPs in *EGFR* and glioma patient OS and PFS

SNP-ID	Genotype	OS		PFS	
		Adjusted HR (95% CI)	*p*	Adjusted HR (95% CI)	*p*
rs17172432	T/T	1	0.512	1	0.729
	C/T	1.121 (0.799–1.571)	0.508	1.018 (0.730–1.418)	0.918
	C/C	2.020 (0.495–8.240)	0.327	1.764 (0.433–7.180)	0.428
rs4947492	A/A	1	0.494	1	0.574
	G/A	1.053 (0.806–1.376)	0.706	0.990 (0.762–1.287)	0.941
	G/G	1.287 (0.848–1.952)	0.235	1.230 (0.808–1.873)	0.335
rs12718945	G/G	1	0.471	1	0.575
	G/T	1.073 (0.820–1.404)	0.606	1.004 (0.771–1.306)	0.978
	T/T	1.297 (0.855–1.965)	0.221	1.239 (0.814–1.886)	0.316
rs730437	A/A	1	0.094	1	0.257
	C/A	1.139 (0.857–1.514)	0.370	1.121 (0.849–1.482)	0.421
	C/C	1.513 (1.041–2.201)	0.030	1.373 (0.942–2.002)	0.100
rs11506105	A/A	1	0.029	1	0.090
	A/G	1.161 (0.877–1.536)	0.297	1.123 (0.854–1.477)	0.406
	G/G	1.680 (1.145–2.466)	0.008	1.542 (1.047–2.272)	0.028
rs3752651	T/T	1	0.064	1	0.051
	C/T	1.101 (0.780–1.554)	0.583	1.037 (0.737–1.460)	0.835
	C/C	5.313 (1.279–22.074)	0.022	5.847 (1.414–24.179)	0.015
rs1468727	T/T	1	0.005	1	0.041
	T/C	1.024 (0.753–1.392)	0.879	1.037 (0.769–1.398)	0.813
	C/C	1.650 (1.158–2.351)	0.006	1.487 (1.049–2.108)	0.026
rs845552	G/G	1	0.027	1	0.039
	G/A	1.297 (0.976–1.724)	0.073	1.253 (0.948–1.656)	0.113
	A/A	1.614 (1.127–2.312)	0.009	1.583 (1.104–2.270)	0.013

OS: Overall survival; PFS: Progression free survival.

HR: Hazard ratio; 95% CI: 95% Confidence interval.

*p* values were calculated using the Wald test.

*p* < 0.05 indicates statistical significance.

## DISCUSSION

In this study, we examined the associations between eight different SNPs in *EGFR* and prognosis of a population of Chinese glioma patients. Our data indicated that age, extent of resection, chemotherapy and five SNPs (rs11506105, rs3752651, rs1468727, rs845552 and rs730437) impacted the survival of glioma patients. In contrast, no significant associations were identified between the eight SNPs in *EGFR* and the prognosis of astrocytoma patients.

Consistent with previous studies, age [18, 19], the extent of resection [20] and chemotherapy [21] were found to be key prognostic factors in glioma patients survival. But not all previous studies, Durmaz, et al. showed that the extent of resection had no significant influence on the survival times of patients with low grade glioma [22]. It is likely that the differing conclusions resulted from the diverse ages and ethnicities of the patients included in the studies, and pathological differences in glioma grade. Although in this study we did not find that WHO grade and radiotherapy were associated glioma patient prognosis, the median OS of the WHO grade I–II glioma patients was longer than that of WHO grade III–IV patients (12 months and 10 months, respectively). These results suggested that WHO grade did have some effect on glioma patient prognosis. It is likely that the samples we collected had some variability. We will therefore verify the results using a larger sample size in future studies., Keime-Guibert et al. simultaneously found that postoperative radiotherapy improved postoperative survival time in elderly patients with high-grade gliomas [23]. This could be explained by the fact that we only analyzed the impact of radiotherapy on glioma patient prognosis and did not perform a comprehensive stratification analysis.

In addition to research focused on the molecular mechanisms underlying glioma development, there is increasing interest in the identification of tumor-related biomarkers. Interestingly, the EGFR gene is the most frequently amplified gene in glioblastoma, and it is a strong prognostic indicator in glioma. Additionally, EGFR-mediated signaling pathways confer growth and survival advantages to tumor cells, promoting a state of continuous and unregulated proliferation. This ultimately results in expansion of the number of malignant cells and a rapid increases in tumor size [24]. Previous studies have shown that EGFR polymorphisms are associated with patient prognosis in a variety of cancers such as head and neck cancer [25], lung cancer [26], colorectal carcinoma [27], breast cancer [28], bladder cancer [29] and esophageal cancer [30]. However, the prognostic value of *EGFR* polymorphisms is not entirely clear. While EGFR amplification and overexpression were previously reported to impact the prognosis of glioma patients, the conclusions of the studies were inconsistent [31–33]. Several clinical and histopathological studies reported that amplification and overexpression of EGFR was associated with a shorter interval to relapse and poor survival in glioblastoma patients [33–35], low-grade glioma patients [36] and anaplastic astrocytoma patients [22, 32]. Nevertheless, in a small study of 107 glioblastoma patients, *EGFR* amplification was not found to be a significant prognostic indicator of OS [37]. The same results were seen in a larger study of 715 glioblastoma patients [38]while the results of another study was inconclusive [31]. Differences in the experimental approaches, sample size, ethnicity of the patient populations or geographic areas may explain the inconsistent results.

Our data indicated that the range of survival times for glioma patients with the C/C genotype of rs3752651 and rs1468727, the G/G genotype of rs11506105 and the A/A genotype of rs845552 were much shorter. It is possible that these SNPs are associated with increased receptor activation, *EGFR* expression or stability, which could increase cancer risk by promoting cell proliferation. Interestingly, one study showed that the G/G genotype of rs845552 was associated with increased survival rin Hispanic and non-Hispanic white breast cancer patients, but no anything association between rs3752651 and the prognosis of breast cancer patients was observed [8]. Similarly, rs3752651 was not significantly associated with non-small cell lung cancer survival in a Chinese population [10]. The prognostic significance of rs4947492 on OS was demonstrated in advanced lung adenocarcinoma patients treated with Gefitinib [39]. However, no significant associations between rs4947492 and glioma patient survival was observed in the current study. This may be explained by the fact that different genetic variants in *EGFR* could have different functional properties that influence prognosis in various cancers. In addition, rs1468727 is located at the intron region indicating it is not likely to affect protein function. The rational explanation may be that these SNPs have a tight linkage with other functional SNPs. While we failed to detect an association between rs730437 and patient prognosis in the univariate survival analysis, multivariate analysis showed that the C/C genotype of rs730437 negatively affected on OS in glioma patients. However, a previous report indicated that the C/C genotype of rs730437 was associated with longer survival time in patients with glioblastoma in the Swedish and Danish [15]. This inconsistency may be explained differences in patient ethnicity. No studies have been published on the complete genetic variance of *EGFR* and glioblastoma prognosis. Thus, future studies of the functions of SNPs in *EGFR* are warranted in order to fully understand their effects on the prognosis of patient with glioma. A previous study found that rs17172432, rs4947492 and rs12718945 were associated with a decreased risk of glioblastoma in a European population [5]. However, the three SNPs were not correlated with the risk of glioma in a Han Chinese population [6]. They had no influence on the prognosis of glioma patients in the present study. Collectively, these data indicate that the three SNPs may have different prognostic effects on glioma patients with differing ethnicities.

It is worth mentioning that there were several inherent limitations in our study. First, the patient cohort included only the Chinese population and the sample size was relatively small. Therefore, our results should be validated in larger patient cohorts consisting of patients from other ethnic groups and geographic areas. Additionally, we could not collect complete and comprehensive information on the clinical pathologic characteristics of the patients. Our data demonstrated that age, extent of resection and chemotherapy are associated with OS and PFS. Finally, the details of the mechanisms underlying the observed associations were beyond the scope of this study. Future studies with more precise clinicopathological data as well as functional studies are required to investigate the role of *EGFR* polymorphisms on glioma patient outcomes. Despite these limitations, the significant association between *EGFR* polymorphisms and prognosis in patients with glioma warrants has demonstrated its potential as a promising therapeutic target in glioma.

In conclusion, our data indicated that age, extent of resection, chemotherapy and five different SNPs (rs11506105, rs3752651, rs1468727 rs845552 and rs730437) in *EGFR* were associated with the prognosis of glioma patients. However, no prominent correlations were observed between the eight SNPs in *EGFR* and the prognosis of astrocytoma patients. Additional studies based on larger sample sizes and patients with different ethnicities are needed to further evaluate the association between genetic polymorphisms in *EGFR* and the prognosis of glioma and astrocytoma patients in larger samples.

## MATERIALS AND METHODS

### Subjects

A total of 269 patients who were diagnosed with glioma at the Department of Neurosurgery, Tangdu Hospital, Fourth Military Medical University, Shaanxi Province (Xi'an, China) between September 2010 and May 2014 were randomly enrolled in this study. The selection criteria were the following: Han Chinese patient with no kinship; recently diagnosed and histologically confirmed to have glioma; no previous history of other cancers; no prior treatment for glioma or prior treatment with chemotherapy or radiotherapy; underwent regular follow-up; peripheral blood samples available. The protocol of this study was approved by the Ethics Committee of Tangdu Hospital, Fourth Military Medical University, Shaanxi Province (Xi'an, China), Northwest University, National Engineering Research Center for Miniaturized Detection Systems and the Department of Anesthesiology, Shaanxi Provincial Tumor Hospital, Xi'an, Shaanxi. All patients gave written informed consent prior to participation in the study.

### SNP selection and genotyping

We selected eight SNPs (rs17172432, rs4947492, rs12718945, rs730437, rs11506105, rs3752651, rs1468727 and rs845552) in EGFR that were found to be associated with glioma risk in European and Han populations [5–7], and influenced the prognosis of both glioblastoma and other cancer patients [10, 15]. Genomic DNAs were extracted through GoldMag-Mini Whole Blood Genomic DNA Purification Kits (GoldMag. Co. Ltd., Xi'an, China) from peripheral blood samples (5 mL) gathered from glioma patients in strict accordance with the manufacturer's protocols. DNA concentrations and purity were evaluated using a spectrophotometer (NanoDrop 2000; Thermo Fisher Scientific, Waltham, MA, USA). We designed polymerase chain reaction (PCR) and extension primers for the SNPs using the Sequenom MassARRAY Assay Design 3.0 software (Sequenom, San Diego, CA, USA). Genotyping of SNPs was designed by using the Sequenom MassARRAY platform with the iPLEX GOLD reagents (Sequenom, San Diego, CA, USA). Finally, we used the Sequenom Typer 4.0 software for data management and analysis.

### Clinical data

For patients, treatment and survival (overall and progression-free) information were collected from a retrospective review of patient medical records or consultation with treating physicians. A standardized questionnaire was used to collect clinical data, including the date of diagnosis, follow-up date(s), gender, age, exact pathology, WHO grade, histologic type, extent of resection, surgical methods, postoperative radiotherapy and chemotherapy. These data were stored electronically using the EpiData3.02 software, and validation, revision and conversion of assigned values were performed to establish the database of glioma patients used for analysis.

### Follow up

We performed the follow-up to analyze the postoperative survival of glioma patients. Follow-up consisted of telephone interviews, outpatient visits, and written communication with patients or their families. Glioma patients who were alive at the time of the analysis were excluded from the study on the day of the final follow-up. Clinical follow-up regarding the genetic status of glioma patients was performed in single-blind fashion with the end point of cardiac death.

### Statistical analyses

All follow-up survey and experimental data were analyzed using SPSS 17.0 (SPSS, Chicago, IL, USA). Survival time was defined as the time between the date of diagnosis and either the date of death (deceased patients) or last contact date (living patients). The OS and PFS were selected as the end evaluation points of this study. OS was measured from the day of surgery until the date of death from any cause or to the date of the last follow-up. PFS was calculated from the date of enrollment to the date of any form of tumor progression or to the last follow-up. The1-year and 3-year survival rates and the MST were determined based on follow-up results. The Kaplan-Meier method was used to estimate PFS and OS. The survival curves were compared using Log-rank tests. Univariate analysis included the following factors: gender, age, WHO grade, extent of resection, radiotherapy, chemotherapy and the eight SNPs. Univariate and multivariable Cox proportional hazard models were used to calculate the crude and adjusted HRs and 95% CIs, respectively. The HRs was adjusted for other factors that could affect glioma outcome such as age, gender, and extent of resection. Two-sided *p* values < 0.05 were considered statistically significant and were calculated using the Wald test.

## SUPPLEMENTARY MATERIALS FIGURES



## References

[1] Ostrom QT, Gittleman H, Farah P, Ondracek A, Chen Y, Wolinsky Y, Stroup NE, Kruchko C, Barnholtz-Sloan JS (2013). CBTRUS statistical report: Primary brain and central nervous system tumors diagnosed in the United States in 2006–2010. Neuro-oncology.

[2] Ricard D, Idbaih A, Ducray F, Lahutte M, Hoang-Xuan K, Delattre JY (2012). Primary brain tumours in adults. Lancet.

[3] Louis DN, Ohgaki H, Wiestler OD, Cavenee WK, Burger PC, Jouvet A, Scheithauer BW, Kleihues P (2007). The 2007 WHO classification of tumours of the central nervous system. Acta neuropathologica.

[4] Yan J, Cheng J, Li H, Liu X, Zheng Y, Wang C, Luo W, Nie Y, Li Z, Pang B, Yang B (2015). Intraoperative high-field magnetic resonance imaging combined with neuronavigation-guided resection of intracranial mesenchymal chondrosarcoma in Broca's area: a rare case report and literature review. International journal of clinical and experimental medicine.

[5] Andersson U, Schwartzbaum J, Wiklund F, Sjostrom S, Liu Y, Tsavachidis S, Ahlbom A, Auvinen A, Collatz-Laier H, Feychting M, Johansen C, Kiuru A, Lonn S (2010). A comprehensive study of the association between the EGFR and ERBB2 genes and glioma risk. Acta oncologica.

[6] Hou WG, Ai WB, Bai XG, Dong HL, Li Z, Zhang YQ, Xiong LZ (2012). Genetic variation in the EGFR gene and the risk of glioma in a Chinese Han population. PloS one.

[7] Wang X, Zhang H, Wang D, Li X (2015). Association of genetic polymorphisms of EGFR with glioma in a Chinese population. Genetic testing and molecular biomarkers.

[8] Connor AE, Baumgartner RN, Baumgartner KB, Pinkston CM, John EM, Torres-Mejia G, Hines LM, Giuliano AR, Wolff RK, Slattery ML (2013). Epidermal growth factor receptor (EGFR) polymorphisms and breast cancer among Hispanic and non-Hispanic white women: the Breast Cancer Health Disparities Study. International journal of molecular epidemiology and genetics.

[9] Poole EM, Curtin K, Hsu L, Kulmacz RJ, Duggan DJ, Makar KW, Xiao L, Carlson CS, Slattery ML, Caan BJ, Potter JD, Ulrich CM (2011). Genetic variability in EGFR, Src and HER2 and risk of colorectal adenoma and cancer. International journal of molecular epidemiology and genetics.

[10] Dong J, Dai J, Shu Y, Pan S, Xu L, Chen W, Wang Y, Jin G, Ma H, Zhang M, Hu Z, Shen H (2010). Polymorphisms in EGFR and VEGF contribute to non-small-cell lung cancer survival in a Chinese population. Carcinogenesis.

[11] Pham D, Kris MG, Riely GJ, Sarkaria IS, McDonough T, Chuai S, Venkatraman ES, Miller VA, Ladanyi M, Pao W, Wilson RK, Singh B, Rusch VW (2006). Use of cigarette-smoking history to estimate the likelihood of mutations in epidermal growth factor receptor gene exons 19 and 21 in lung adenocarcinomas. Journal of clinical oncology.

[12] Jami MS, Hemati S, Salehi Z, Tavassoli M (2008). Association between the length of a CA dinucleotide repeat in the EGFR and risk of breast cancer. Cancer investigation.

[13] Yano S, Kondo K, Yamaguchi M, Richmond G, Hutchison M, Wakeling A, Averbuch S, Wadsworth P (2003). Distribution and function of EGFR in human tissue and the effect of EGFR tyrosine kinase inhibition. Anticancer research.

[14] Brandt B, Meyer-Staeckling S, Schmidt H, Agelopoulos K, Buerger H (2006). Mechanisms of egfr gene transcription modulation: relationship to cancer risk and therapy response. Clinical cancer research.

[15] Sjostrom S, Andersson U, Liu Y, Brannstrom T, Broholm H, Johansen C, Collatz-Laier H, Henriksson R, Bondy M, Melin B (2010). Genetic variations in EGF and EGFR and glioblastoma outcome. Neuro-oncology.

[16] Waha A, Baumann A, Wolf HK, Fimmers R, Neumann J, Kindermann D, Astrahantseff K, Blumcke I, von Deimling A, Schlegel U (1996). Lack of prognostic relevance of alterations in the epidermal growth factor receptor-transforming growth factor-alpha pathway in human astrocytic gliomas. Journal of neurosurgery.

[17] Bienkowski M, Piaskowski S, Stoczynska-Fidelus E, Szybka M, Banaszczyk M, Witusik-Perkowska M, Jesien-Lewandowicz E, Jaskolski DJ, Radomiak-Zaluska A, Jesionek-Kupnicka D, Sikorska B, Papierz W, Rieske P (2013). Screening for EGFR amplifications with a novel method and their significance for the outcome of glioblastoma patients. PloS one.

[18] Gorlia T, Wu W, Wang M, Baumert BG, Mehta M, Buckner JC, Shaw E, Brown P, Stupp R, Galanis E, Lacombe D, van den Bent MJ (2013). New validated prognostic models and prognostic calculators in patients with low-grade gliomas diagnosed by central pathology review: a pooled analysis of EORTC/RTOG/NCCTG phase III clinical trials. Neuro-oncology.

[19] Mikheev AM, Ramakrishna R, Stoll EA, Mikheeva SA, Beyer RP, Plotnik DA, Schwartz JL, Rockhill JK, Silber JR, Born DE, Kosai Y, Horner PJ, Rostomily RC (2012). Increased age of transformed mouse neural progenitor/stem cells recapitulates age-dependent clinical features of human glioma malignancy. Aging cell.

[20] Sanai N, Berger MS (2011). Extent of resection influences outcomes for patients with gliomas. Revue neurologique.

[21] Stewart LA (2002). Chemotherapy in adult high-grade glioma: a systematic review and meta-analysis of individual patient data from 12 randomised trials. Lancet.

[22] Durmaz R, Vural M, Isildi E, Cosan E, Ozkara E, Bal C, Ciftci E, Arslantas A, Atasoy MA (2008). Efficacy of prognostic factors on survival in patients with low grade glioma. Turkish neurosurgery.

[23] Keime-Guibert F, Chinot O, Taillandier L, Cartalat-Carel S, Frenay M, Kantor G, Guillamo JS, Jadaud E, Colin P, Bondiau PY, Menei P, Loiseau H, Bernier V (2007). Radiotherapy for glioblastoma in the elderly. The New England journal of medicine.

[24] Tabernero J (2007). The role of VEGF and EGFR inhibition: implications for combining anti-VEGF and anti-EGFR agents. Molecular cancer research.

[25] Bandres E, Barricarte R, Cantero C, Honorato B, Malumbres R, Zarate R, Alcalde J, Garcia-Foncillas J (2007). Epidermal growth factor receptor (EGFR) polymorphisms and survival in head and neck cancer patients. Oral oncology.

[26] Sasaki H, Okuda K, Shimizu S, Takada M, Kawahara M, Kitahara N, Okumura M, Matsumura A, Iuchi K, Kawaguchi T, Kubo A, Kawano O, Yukiue H (2009). EGFR R497K polymorphism is a favorable prognostic factor for advanced lung cancer. Journal of cancer research and clinical oncology.

[27] Wang WS, Chen PM, Chiou TJ, Liu JH, Lin JK, Lin TC, Wang HS, Su Y (2007). Epidermal growth factor receptor R497K polymorphism is a favorable prognostic factor for patients with colorectal carcinoma. Clinical cancer research.

[28] Jin Q, Hemminki K, Enquist K, Lenner P, Grzybowska E, Klaes R, Henriksson R, Chen B, Pamula J, Pekala W, Zientek H, Rogozinska-Szczepka J, Utracka-Hutka B (2005). Vascular endothelial growth factor polymorphisms in relation to breast cancer development and prognosis. Clinical cancer research.

[29] Mason RA, Morlock EV, Karagas MR, Kelsey KT, Marsit CJ, Schned AR, Andrew AS (2009). EGFR pathway polymorphisms and bladder cancer susceptibility and prognosis. Carcinogenesis.

[30] Lee JM, Yang SY, Yang PW, Shun CT, Wu MT, Hsu CH, Lin CC, Cheng JC, Wang YH, Chuang TH, Chen JS, Hsu HH, Huang PM (2011). Polymorphism in epidermal growth factor receptor intron 1 predicts prognosis of patients with esophageal cancer after chemoradiation and surgery. Annals of surgical oncology.

[31] Bouvier-Labit C, Chinot O, Ochi C, Gambarelli D, Dufour H, Figarella-Branger D (1998). Prognostic significance of Ki67, p53 and epidermal growth factor receptor immunostaining in human glioblastomas. Neuropathology and applied neurobiology.

[32] Smith JS, Tachibana I, Passe SM, Huntley BK, Borell TJ, Iturria N, O'Fallon JR, Schaefer PL, Scheithauer BW, James CD, Buckner JC, Jenkins RB (2001). PTEN mutation, EGFR amplification, and outcome in patients with anaplastic astrocytoma and glioblastoma multiforme. Journal of the National Cancer Institute.

[33] Ruano Y, Ribalta T, de Lope AR, Campos-Martin Y, Fiano C, Perez-Magan E, Hernandez-Moneo JL, Mollejo M, Melendez B (2009). Worse outcome in primary glioblastoma multiforme with concurrent epidermal growth factor receptor and p53 alteration. American journal of clinical pathology.

[34] Shinojima N, Tada K, Shiraishi S, Kamiryo T, Kochi M, Nakamura H, Makino K, Saya H, Hirano H, Kuratsu J, Oka K, Ishimaru Y, Ushio Y (2003). Prognostic value of epidermal growth factor receptor in patients with glioblastoma multiforme. Cancer research.

[35] Hurtt MR, Moossy J, Donovan-Peluso M, Locker J (1992). Amplification of epidermal growth factor receptor gene in gliomas: histopathology and prognosis. Journal of neuropathology and experimental neurology.

[36] Andersson U, Guo D, Malmer B, Bergenheim AT, Brannstrom T, Hedman H, Henriksson R (2004). Epidermal growth factor receptor family (EGFR, ErbB2–4) in gliomas and meningiomas. Acta neuropathologica.

[37] Quan AL, Barnett GH, Lee SY, Vogelbaum MA, Toms SA, Staugaitis SM, Prayson RA, Peereboom DM, Stevens GH, Cohen BH, Suh JH (2005). Epidermal growth factor receptor amplification does not have prognostic significance in patients with glioblastoma multiforme. International journal of radiation oncology, biology, physics.

[38] Ohgaki H, Dessen P, Jourde B, Horstmann S, Nishikawa T, Di Patre PL, Burkhard C, Schuler D, Probst-Hensch NM, Maiorka PC, Baeza N, Pisani P, Yonekawa Y (2004). Genetic pathways to glioblastoma: a population-based study. Cancer research.

[39] Zhang L, Yuan X, Chen Y, Du XJ, Yu S, Yang M (2013). Role of EGFR SNPs in survival of advanced lung adenocarcinoma patients treated with Gefitinib. Gene.

