# Supplementary material for: Effect of epidermal growth factor receptor gene polymorphisms on prognosis in glioma patients
Source: Oncotarget. 2016 Jul 18;7(39):63054–64. doi: 10.18632/oncotarget.10666 (PMC5325346; doi:10.18632/oncotarget.10666)
Supplement: Supplementary file 1 [file oncotarget-07-63054-s001.pdf]

Effect of epidermal growth factor receptor gene polymorphisms on prognosis in glioma patients

Supplementary Materials

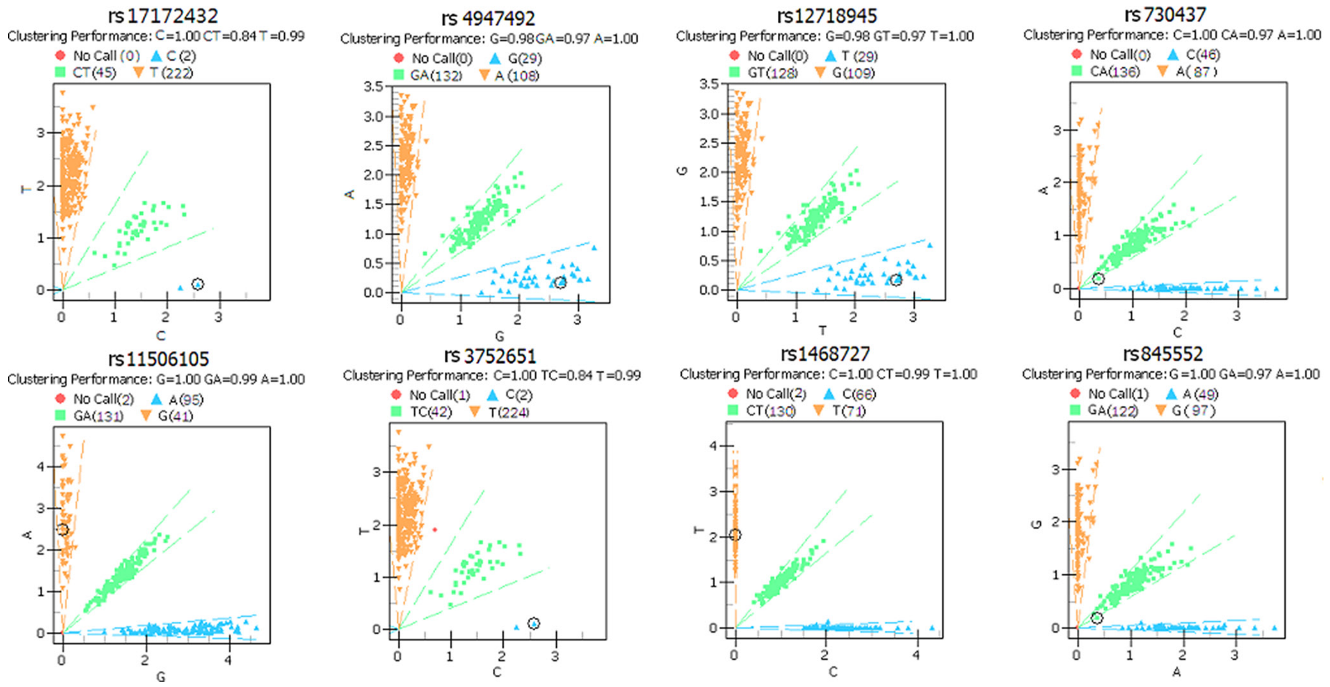

Supplementary Figure S1: The genotyping results of eight SNPs (rs17172432, rs4947492, rs12718945, rs730437, rs11506105, rs3752651, rs1468727 and rs845552)
